# 双特异性T细胞衔接器治疗急性淋巴细胞白血病指导原则（2022年版）

**DOI:** 10.3760/cma.j.issn.0253-2727.2022.06.002

**Published:** 2022-06

**Authors:** 中国临床肿瘤学会白血病专家委员会 中国临床肿瘤学会淋巴瘤专家委员会 中华医学会血液学分会

前体B细胞急性淋巴细胞白血病（Precursor B-cell acute lymphoblastic leukemia, BCP-ALL）是一种进展迅速的白血病，表现为骨髓、外周血和其他器官存在过多B 淋巴细胞母细胞。按照美国癌症研究院2020年的数据，美国每年大约有6 150人被诊断为急性淋巴细胞白血病（ALL），死亡人数大约为1 520人[1]。得益于高强度化疗方案的使用，成人新诊断ALL患者的长期生存率近30年已经提高至30％～50％，但复发或难治性（R/R）ALL患者的中位总生存（OS）时间仅为2～6个月，3～5年生存率低于10％。成人R/R ALL亟需更有效的治疗方法[2]。

肿瘤的免疫治疗旨在利用免疫系统治疗肿瘤，双特异性抗体（Bispecific Antibody, BsAb）被设计用于识别和结合两种不同的抗原或表位。在过去的几十年里，BsAb已经在肿瘤治疗的背景下发展起来，特别是用于治疗血液系统的恶性肿瘤。到目前为止，已有一百多种不同类型的BsAb[3]。双特异性T细胞衔接器（Bispeific T cell Engager，BiTE）作为BsAb中较为成熟的技术平台，特异性地与T细胞表面CD3和肿瘤细胞表面特异性抗原结合，通过募集和激活肿瘤附近的多克隆T细胞群，而不需要共刺激因子或主要组织相容性复合体（MHC）的识别，对肿瘤细胞进行持续地杀伤裂解[4]。

贝林妥欧单抗是第一个在全球范围内被批准的BiTE分子，靶向B细胞上的CD19表面抗原[5]。贝林妥欧单抗于2014年12月3日被FDA批准用于治疗成人Ph阴性的R/R BCP-ALL，成为第一个获得FDA加速审批上市的BiTE。成熟的BiTE技术和有效的靶点选择使适应证不断拓展，在成人R/R BCP-ALL适应证后，FDA于2017年7月批准其将适应证扩展至包含Ph阳性的成人及儿童R/R BCP-ALL，并于2018年3月加速批准用于第1次完全缓解（CR_1_）或CR_2_伴微小残留病（MRD）阳性的成人及儿童BCP-ALL[6]。

一、BiTE概述

1. BiTE技术平台：随着BsAb的分子模型和制备技术不断发展，BiTE技术由于成药稳定性、制备工艺、治疗效果等方面的前景，逐渐被广泛应用于肿瘤治疗领域的研发。BiTE抗体结构是由两个单链可变区（single-chain variable fragment, scFv）通过灵活的肽链连接而成，一侧结合域可识别肿瘤表达抗原（如CD19、CD33、BCMA等），另一侧通常特异性识别CD3，即T细胞受体复合物的成分之一，从而可以衔接T细胞和肿瘤靶细胞，引起T细胞活化并杀伤肿瘤靶细胞，因此是针对各种肿瘤的疗法，并且接受再治疗仍可能有效。与其他BsAb相比，BiTE具有相对分子质量小、肿瘤穿透作用强、单链可变区之间的连接柔韧性更好等特点[7]。

2. BiTE及贝林妥欧单抗的作用机制：当BiTE分子与细胞毒性T细胞和肿瘤细胞结合，T细胞即开始增殖，通过增加效应细胞的数量来增强BiTE的治疗效力，恶性肿瘤细胞的裂解即被触发。由于这一过程不需要共刺激分子或典型的MHC机制，BiTE分子可以与任何T细胞结合[5]。

贝林妥欧单抗（CD19×CD3 BiTE）是基于BiTE技术平台开发的免疫药物，是首个也是目前唯一获批的用于治疗成人及儿童ALL的CD3×CD19双抗。贝林妥欧单抗作为一种双特异性CD19导向的CD3 T细胞衔接分子（鼠源性重组单链），与B系细胞表面的CD19和T细胞表面的CD3结合，从而介导T细胞与肿瘤细胞间免疫突触的形成、上调细胞黏附分子、产生细胞水解蛋白、释放炎性细胞因子并促进T细胞增殖，从而定向裂解CD19阳性肿瘤细胞，可用于B系淋巴细胞恶性血液肿瘤的治疗[8]。

3. 贝林妥欧单抗的药代动力学特点：成人患者贝林妥欧单抗的药代动力学特征呈线性。连续静脉输注后，1 d内达到稳态血清浓度（Css），并随时间推移保持稳定。治疗R/R ALL时，在临床剂量9 µg/d或28 µg/d时，平均（SD）Css分别为228（356）pg/ml和616（537）pg/ml。贝林妥欧单抗的终末相平均分布容积（Vz）为4.52 L，与血浆容积相似，表明其主要分布在血管。该药代谢途径尚不清楚，推测可能与其他蛋白质疗法相似，即通过分解代谢途径降解为小分子肽和氨基酸。贝林妥欧单抗的平均半衰期为2.11 h，平均全身清除率估算为3.11 L/h，因此需连续静脉给药28 d以维持有效稳态浓度[9]。

在既往同时纳入儿童和成人患者的研究中发现，贝林妥欧单抗的清除率在儿童患者（<18岁）中随体表面积（body surface area, BSA）增加而增加，而在成人患者中，人口统计学特征（如体重、BSA、年龄、性别）、疾病类型、基线ALT/AST水平等均与贝林妥欧单抗的清除率无明显相关性，并且对于轻度或中度肾功能不全的患者无需调整剂量。因此在低体重患者中采用基于BSA的给药方案，而成人≥45 kg患者给予固定剂量的给药方案[9]–[10]。

4. 贝林妥欧单抗的药效动力学特点：贝林妥欧单抗的药效学反应表现为T细胞活化和再分布、外周血B细胞减少和一过性的细胞因子升高。

在开始连续输注贝林妥欧单抗后，外周CD3^+^ T细胞于1～2 d内下降至极低水平，随后在7～14 d内迅速恢复至基线，这种现象称为“再分布”。在持续输注2～3周后，大部分患者的T细胞数量扩增至基线的2倍以上，提示贝林妥欧单抗对T细胞有扩增作用。

与T细胞的改变相反，外周血B细胞在输注贝林妥欧单抗后的平均2 d内即迅速下降至<1个细胞/µl，且在整个治疗周期中持续为无法检测到的水平。在治疗间歇期的2周未观察到外周血B细胞计数恢复。

对细胞因子IL-2、IL-4、IL-6、IL-8、IL-10、IL-12、TNF-α和TNF-γ进行了测定，结果发现IL-6、IL-10和TNF-γ水平升高。在开始本品输注后2 d内观察到细胞因子升高幅度最大，在24至48 h内返回至基线。与第1治疗周期的前48 h相比，后续治疗周期中发生细胞因子水平升高的患者更少，且强度更低，因此贝林妥欧单抗的免疫治疗相关不良反应多发生在第1治疗周期的7～10 d内[10]–[12]。

二、贝林妥欧单抗治疗成人ALL的临床研究

在临床研究中，贝林妥欧单抗不仅在成人及儿童R/R ALL的治疗中显示出了比化疗优越的疗效及安全性，在获得CR伴MRD阳性患者中同样表现出了很好的清除MRD疗效，显著提高了患者的CR/CR伴部分血液学缓解（CRh）率及MRD阴性率，从而降低了疾病进展和死亡风险，延长了患者的生存期。

1. R/R BCP-ALL：

（1）Ph阴性R/R BCP-ALL：为验证贝林妥欧单抗在Ph阴性R/R BCP-ALL患者中的疗效及安全性，Ⅱ期临床研究入组了189例成人原发性难治或首次缓解后1年内复发、或接受异基因造血干细胞移植（allo-HSCT）后12个月内复发、或在首次或更多次挽救治疗后复发或难治的Ph阴性BCP-ALL患者，均接受贝林妥欧单抗单药治疗。2个周期治疗后，43％的患者达到CR/CRh，其中CR率为33％，40％的CR/CRh患者后续桥接了HSCT，2％的患者发生3级细胞因子释放综合征（cytokine release syndrome, CRS），13％的患者出现3～4级神经系统毒性，入组的患者无一例发生致命性神经系统毒性事件[13]。

在Ⅱ期临床研究基础上，为进一步比较贝林妥欧单抗与目前临床常用治疗即多药化疗方案的疗效及安全性，开展了一项国际多中心、随机对照Ⅲ期研究（TOWER研究）。研究共入组405例患者，以2∶1比例随机分配至贝林妥欧单抗组或标准化疗组（研究者选择以下四种化疗方案之一：FLAG方案、大剂量阿糖胞苷为基础的方案、大剂量甲氨蝶呤为基础的方案、氯法拉滨为基础的方案），所有患者先接受2个周期诱导治疗，达缓解者继续接受3个周期贝林妥欧单抗巩固治疗及后续1年的维持治疗。两组中将试验方案作为首次挽救治疗（first salvage, S1）的患者比例相似（42.1％对48.5％），且均以高肿瘤负荷患者为主（原始细胞≥50％患者比例：74.2％对77.6％）。后续根据研究者判定选择桥接HSCT或接受其他抗肿瘤治疗，贝林妥欧单抗组中位治疗周期数为2，化疗组中位接受1个周期治疗。中位随访时间11.8个月，贝林妥欧单抗组的CR率（CR/CRh/CRi：44％对5％，*P*<0.001）、MRD阴性率（76％对48％）及生存获益［中位OS期：7.7个月对4.0个月，*HR*＝0.71，*P*＝0.01；6个月无事件生存（EFS）率：31％对12％，*HR*＝0.55，*P*<0.001］均显著高于标准化疗组[2]。安全性方面在根据药物暴露进行校正后对比显示，贝林妥欧单抗组≥3级不良事件的发生率显著低于标准化疗组（10.73对45.27事件/患者年，*P*<0.001）[14]，≥3级的CRS及神经毒性事件发生率为4.9％和9.4％，值得注意的是，化疗组8.3％的患者出现3级及以上神经毒性事件。鉴于从贝林妥欧单抗组观察到的显著获益，在入组病例死亡数达到预估的75％时，独立数据和安全监测委员会建议提前结束了该研究。

进一步亚组分析显示，接受研究方案作为S1的167例患者中，贝林妥欧单抗较化疗更显著地改善12周内的CR率（CR/CRh/CRi：51％对36.5％，*P*＝0.069），并延长中位OS期（11.1个月对5.5个月，*HR*＝0.59，*P*＝0.016），而将试验用药作为二次及以上挽救治疗（S2+）的患者CR/CRh/CRi率为39.5％和14.1％（*P*<0.001）[15]。针对后续接受了allo-HSCT患者的亚组分析显示，将贝林妥欧单抗作为S1获得MRD缓解的患者，桥接HSCT至1年时OS率100％，18个月OS率80％，提示早期使用贝林妥欧单抗以及后续桥接HSCT可获益更多[16]。

在中国发起的单臂3期研究（316研究）显示，截至2019年4月的中期报告分析了90例与TOWER研究一致的R/R BCP-ALL患者，骨髓原始细胞比例≥50％患者占77.8％，中位接受贝林妥欧单抗治疗时间为49.8 d，两个治疗周期内的CR/CRh率为45.6％，其中MRD缓解率为82.9％，中位OS期9.2个月，中位无复发生存（RFS）期4.3个月，3级及以上CRS发生率为6.7％，3级及以上神经毒性事件发生率为5.6％，无新的安全性风险事件发生，药代动力学结果与全球研究数据一致，验证了在中国人群中的疗效及安全性[17]。

（2）Ph阳性R/R BCP-ALL：酪氨酸激酶抑制剂（TKI）是Ph阳性ALL患者的有效治疗手段，但随着TKI的广泛应用，TKI难治及复发的患者数量增加，为验证贝林妥欧单抗对此类患者的疗效，开展了一项Ⅱ期临床研究（ALCANTARA研究），将贝林妥欧单抗单药用于既往接受过至少一种二代及以上TKI治疗后复发、难治或无法耐受，或对伊马替尼无法耐受或难治的成人Ph阳性BCP-ALL患者，结果显示45例患者中在前两个治疗周期达到CR/CRh的患者占36％（包括10例伴T315I突变的患者），其中88％的CR/CRh患者进一步获得MRD完全缓解，44％的患者后续接受了allo-HSCT，只有3例发生1～2级CRS，无患者发生≥3级CRS，3级神经系统毒性的发生率为6.7％，无4～5级神经系统毒性事件发生。显示了贝林妥欧作为新型免疫治疗亦可用于TKI治疗无效的Ph阳性BCP-ALL患者[18]。

2. MRD清除：MRD是与ALL复发显著相关的预测因子，30％～50％的成人ALL患者在诱导化疗达到CR后，流式细胞术或PCR检测MRD仍然为阳性，因此BLAST研究即为评估贝林妥欧单抗用于CR伴MRD≥10^−3^的成人BCP-ALL患者，观察清除MRD的疗效和安全性的Ⅱ期临床研究。研究共入组116例患者，75％的患者基线MRD≥10^−2^，经过1个周期贝林妥欧单抗治疗后88％的患者获得MRD缓解，其中80％为MRD完全缓解。全部患者的中位OS期为36.5个月，RFS期为18.9个月，67％的持续CR患者后续桥接了HSCT。CRS总体发生率为3％（均为3级及以下），且均发生于治疗的第一周期，3级及以上神经毒性事件发生率为13％，不良事件发生率随治疗周期增加逐渐下降[19]。在延长随访时间至中位59.8个月后，经贝林妥欧单抗治疗获得MRD完全缓解患者的生存获益更加突出：中位OS期未达到。其中，CR_1_的患者相比于复发后治疗达到CR（CR_2+_）的患者使用贝林妥欧单抗治疗中位OS期更长（未达到对38.8个月），后续未接受HSCT患者仍获得中位56.4个月的长期生存。提示尽早使用贝林妥欧单抗作为MRD清除治疗的生存获益更佳，并且为无法移植的患者如老年或无合适供者的患者带来了长期生存的可能[20]。

三、贝林妥欧单抗治疗儿童ALL的临床研究

1. 儿童关键性Ⅰ/Ⅱ期临床研究：205研究为在<18岁的R/R BCP-ALL患者中开展的Ⅰ/Ⅱ期临床研究，49例患者入组Ⅰ期研究进行毒性限制剂量探索，儿童的最大耐受剂量为15 µg·m^−2^·d^−1^，即第1个治疗周期的前7 d剂量为5 µg·m^−2^·d^−1^，之后均接受15 µg·m^−2^·d^−1^的贝林妥欧单抗直至治疗结束。其中对Ⅰ期试验中接受了推荐剂量治疗的26例及入组Ⅱ期试验的共70例患者进行疗效及安全性分析显示，入组的患者绝大多数为肿瘤负荷高（74％为骨髓原始细胞比例≥50％）、移植后复发（57％）及至末次治疗间隔<6个月的患者（71％），两个治疗周期内的CR率为39％，其中74％的患者在第1个治疗周期获得CR，MRD缓解率为52％，中位生存期为7.5个月，而在这些肿瘤负荷较高的患者中3级及以上CRS（5.7％）和神经毒性（4％）发生率较成人患者更低，提示贝林妥欧单抗在儿童患者中应用相对安全[21]。

鉴于贝林妥欧单抗在205研究中显示的疗效及安全性，欧洲研究者在贝林妥欧单抗儿科适应证获批前，开展了一项在儿童BCP-ALL中的扩展用药（同情给药）研究即RIALTO研究，入组110例R/R及骨髓原始细胞<5％伴MRD≥10^−3^（12例）的儿童BCP-ALL患者，在骨髓原始细胞≥5％的98例患者中，两个治疗周期内的CR率为59％，获得CR患者的MRD缓解率为79％；而在原始细胞<5％伴MRD阳性的患者中，在两个治疗周期内MRD缓解率为92％，所有获得CR的患者中65％后续接受了HSCT。所有患者的中位OS期为13.1个月，获得MRD缓解的患者生存期更长达21.3个月。安全性方面，≥3级CRS（2.8％）和神经毒性事件（5.5％）发生率与205研究基本一致，进一步验证了贝林妥欧单抗在儿童患者中应用的安全性及疗效[22]。

2. 儿童关键性Ⅲ期临床研究：allo-HSCT是首次复发的儿童BCP-ALL的重要治疗手段，但中高危患儿如早期复发者常因化疗相关不良反应（严重感染等）无法接受移植，以及再诱导治疗后无法获得MRD阴性而降低移植预后，因此为评估贝林妥欧单抗与化疗在首次复发的中高危患儿中用于巩固治疗对预后的影响，国外先后发起了两项多中心的Ⅲ期临床研究，近期均公布了研究结果。

215研究是一项多中心随机对照Ⅲ期研究，共纳入108例年龄>28 d且≤18岁的高危首次复发的B-ALL患者，经诱导化疗及两个周期巩固治疗达到形态学CR（M_1_骨髓，原始细胞<5％）或M_2_骨髓（5％≤原始细胞<25％），1∶1随机给予贝林妥欧单抗或化疗作为第3周期巩固治疗。贝林妥欧单抗组的MRD缓解率显著高于化疗组（90％对54％），两组获得CR_2_的患者后续桥接allo-HSCT的比例分别为88.9％和70.4％；贝林妥欧单抗组EFS期显著长于化疗组，24个月EFS率分别为66.2％和27.1％（*HR*＝0.33，*P*<0.001）；贝林妥欧单抗组复发率显著低于化疗组（24.1％对53.7％，*HR*＝0.24，95％*CI* 0.13～0.46），两组死亡率分别为14.8％和29.6％，OS的*HR*为0.43（95％*CI* 0.18～1.01）。安全性方面贝林妥欧单抗优于化疗，3级及以上不良事件发生率分别为57.4％和82.4％，贝林妥欧单抗组3级及以上CRS（1.9％）及神经毒性事件（5.6％）发生率均较低。鉴于贝林妥欧单抗组显示出的显著获益，该研究被提前终止[23]。

另一项由儿童肿瘤协会（Children's Oncology Group, COG）在欧美155家医院中针对中-高危首次复发的B-ALL患者开展的Ⅲ期临床研究（COG AALL-1331研究），共纳入了208例1～30岁受试者，所有患者在接受4周的诱导化疗后随机分配至贝林妥欧单抗或化疗组继续接受2个周期的巩固治疗。在第1个周期巩固治疗结束后，贝林妥欧单抗组的MRD阴性率显著高于化疗组（75％对32％，*P*<0.001），有更高比例的患者后续接受了allo-HSCT（70％对43％，*P*<0.001）；中位随访2.9年，贝林妥欧单抗组与化疗组的2年无病生存（DFS）率分别为54.4％和39.0％（*HR*＝0.7，*P*＝0.03），2年OS率为71.3％和58.4％（*HR*＝0.62，*P*＝0.02）。贝林妥欧单抗组≥3级不良事件发生率低于化疗组（61％对83％），累积感染和发热伴粒细胞缺乏发生率分别为15％和5％，化疗组为65％和58％，且贝林妥欧单抗组所有治疗相关不良事件均可逆，且无不良事件相关死亡，化疗组有5例患者死亡[24]。虽然COG AALL-1331研究DFS未达到预设的优效界值*P*＝0.004，但由于贝林妥欧单抗组显示出更长的DFS、OS时间，更少的毒性和更深的MRD缓解，数据和安全监测委员会确信其可为患者带来获益，提前终止研究。贝林妥欧单抗在高危首次复发儿童B-ALL治疗的最佳时机仍在向前探索，同时近期一项评估贝林妥欧单抗单药或联合纳武利尤单抗用于早期复发B-ALL再诱导后伴MRD的清除治疗研究也正在进行[25]。

四、其他贝林妥欧单抗在ALL中的临床探索

免疫治疗与小分子靶向药及化疗的联合探索近年来受到关注，贝林妥欧单抗在B-ALL领域已有与TKI及化疗方案联合用于新诊断BCP-ALL的研究结果。

贝林妥欧单抗与TKI联合治疗新诊断Ph阳性BCP-ALL的疗效及安全性在一项Ⅱ期研究中已得到了印证。研究入组了63例24～82岁患者，诱导治疗为7 d激素预治疗序贯达沙替尼（140 mg×85 d）联合泼尼松（24 d），巩固治疗阶段接受达沙替尼联合贝林妥欧单抗至少2个周期，诱导治疗后98％患者获得血液学CR，分子学缓解率29％，经巩固治疗后分子学缓解为60％，第四周期达81％，p190和p210阳性患者在经过2个周期贝林妥欧单抗治疗后的分子学缓解率差异无统计学意义。中位随访18个月OS率95％，DFS为88％，38％的患者后续桥接了allo-HSCT[26]。

另一项2020年ASH会议上公布的针对成人新诊断Ph阴性B-ALL的Ⅱ期临床研究显示，在接受Hyper-CVAD方案4个周期化疗后序贯4个治疗周期标准剂量的贝林妥欧单抗，维持治疗为POMP方案与贝林妥欧单抗交替，28例入组患者100％获得CR，其中82％在第1个治疗周期时获得，MRD阴性率为97％（第1个周期结束时为87％）。中位随访22个月，2年持续缓解率为79％，2年OS率为86％，桥接allo-HSCT比例达35％。CRS发生率为10％，均为2～3级，神经系统毒性发生率为41％，仅1例患者因贝林妥欧单抗相关不良反应导致治疗终止[27]。两项研究证实贝林妥欧单抗与靶向药或化疗联合用于新诊断成人B-ALL患者均有较好的疗效及安全性。

五、贝林妥欧单抗治疗B-ALL的临床应用推荐

基于以上贝林妥欧单抗在成人及儿童患者中的研究数据，各国指南针对贝林妥欧单抗在B-ALL中的应用做了相关推荐。

1. 成人B-ALL：2016年欧洲肿瘤内科学会（ESMO）指南[28]指出，成人及老年ALL仍需在现有化疗/移植条件下改善治疗，B-ALL的抗体治疗中贝林妥欧单抗在MRD阳性及R/R ALL中均已证实有效。

2021年第1版美国国立综合癌症网络（NCCN）指南[1]中，青少年和年轻成人（AYA）以及成人Ph阴性ALL在诱导治疗结束获得CR伴MRD阳性的患者，优先推荐贝林妥欧单抗治疗，Ph阳性患者优先推荐贝林妥欧单抗联合或不联合TKI，后续考虑桥接allo-HSCT；R/R ALL不论Ph阴性或阳性，均推荐贝林妥欧单抗治疗并后续考虑桥接allo-HSCT，其中Ph阴性为一类优先推荐。

中国2021年CSCO指南[29]中同样推荐诱导治疗后CR伴MRD阳性B-ALL患者使用贝林妥欧单抗，R/R Ph阴性患者推荐贝林妥欧单抗治疗。

治疗推荐：①对于复发难治患者，越早用贝林妥欧单抗生存获益越多，推荐首次挽救治疗即选择贝林妥欧单抗，以达到更快更深的缓解，后续桥接allo-HSCT可获得更长生存。②对于经诱导治疗获得CR伴MRD阳性的患者，不论CR_1_或CR_2_，均推荐以贝林妥欧单抗进行MRD清除治疗，以获得更持久的缓解和延长生存。

2. 儿童B-ALL：2021年第2版NCCN指南[30]中，Ph阴性或Ph-like B-ALL在诱导治疗后巩固治疗阶段MRD阳性患者在经临床试验或高强度化疗后仍未转阴者推荐贝林妥欧单抗治疗，Ph阳性B-ALL诱导治疗后高危患者推荐贝林妥欧单抗，后续桥接allo-HSCT；对难治复发患者，推荐首次复发的B-ALL，早期复发再次诱导治疗后无论MRD状态及allo-HSCT后复发者，以及难治和多次复发患者接受贝林妥欧单抗治疗。

2018年版中国儿童ALL诊疗规范在分子靶向药物治疗中也指出贝林妥欧单抗为B系ALL患儿带来了激动人心的治疗反应[31]。

治疗推荐：难治复发患者，越早使用贝林妥欧单抗获益越多，推荐首次复发者的挽救治疗和早期复发、诱导后MRD阳性即中高危患者的巩固治疗，后续桥接allo-HSCT可获得更长生存。

六、BiTE临床应用的管理

1. 贝林妥欧单抗推荐用药方法：贝林妥欧单抗的剂量选择根据体重是否超过45 kg进行划分，≥45 kg的患者接受固定剂量给药，<45 kg的患者根据BSA计算剂量。难治及复发患者需在第1治疗周期的第1～7天从低剂量起始，体重≥45 kg者给予固定剂量9 µg/d，<45 kg者根据BSA给予5 µg·m^−2^·d^−1^；第8天起至第28天增加至足剂量即≥45 kg者28 µg/d，<45 kg者15 µg·m^−2^·d^−1^。用于MRD阳性患者无需剂量爬坡，起始即给予足剂量28 µg/d（≥45 kg）或15 µg·m^−2^·d^−1^（<45 kg）。如治疗期间出现中断给药（如因不良事件发生），未超过7 d可继续该周期治疗直至输注28 d，中断给药超过7 d则需重新开始新的治疗周期[9]–[10]。

上述复发或难治ALL关键临床研究中，贝林妥欧单抗中位治疗1～2个周期，所有获得CR的患者都在2个周期内实现，真实世界的用药结局[32]–[41]与临床研究一致。达到缓解后可根据患者情况桥接移植或后续治疗。

2. 早期用药及注意事项：在开始贝林妥欧单抗治疗前对患者的肿瘤负荷进行评估以避免严重CRS的发生，包括骨髓原始细胞比例≥50％或外周血原始细胞计数>15 000/µl。针对高肿瘤负荷的患者，在开始贝林妥欧单抗治疗前需给予地塞米松进行预先治疗（不超过24 mg/d），并在每个治疗周期第1次给药前1 h、升高剂量前以及中断治疗4 h后重启输注时，预先给予20 mg地塞米松[2],[32]。

3. 疗效评估：因贝林妥欧单抗的治疗周期不同于常规化疗的治疗周期，在临床试验中骨髓穿刺的监测时间为筛选期（治疗前）、每个治疗周期结束时（第29天±8天）以及长期随访期每3个月±2周，以评估细胞形态学缓解及MRD情况[2]。贝林妥欧单抗治疗过程中应定期根据相关疾病指南进行疗效评估。通常治疗后1～2个月即可见相关指标的改善。

4. 免疫相关不良反应管理：上述研究已证实贝林妥欧单抗总体安全性可管理，无论是≥3级不良反应还是血液学不良反应均低于化疗，而BiTE分子在临床应用中值得关注的是CRS及神经毒性，随着包括CAR-T、免疫检查点抑制剂、BiTE等在内的免疫治疗兴起，国内外对免疫治疗相关不良反应的管理也日趋成熟[32]。

（1）CRS：CRS的表现主要包括发热、头痛、恶心、乏力、低血压、丙氨酸转氨酶（ALT）升高、天冬氨酸转氨酶（AST）升高、总胆红素升高以及弥散性血管内凝血（DIC）等。接受免疫治疗后的CRS的表现可能与输液反应、毛细血管渗漏综合征（CLS）和噬血细胞性组织细胞增生症/巨噬细胞激活综合征（MAS）的表现重叠[42]，应高度关注。

在TOWER研究中，CRS发生率为14.2％，≥3级CRS发生率为4.9％，因CRS导致贝林妥欧单抗治疗的中断率为5％，导致治疗终止率为1％。CRS发生中位时间为输注开始后第2天，CRS消退中位时间为第5天。大多数CRS是可逆的，在贝林妥欧单抗给药前通过识别高危患者、地塞米松预先用药及逐步增加剂量的给药方式进行预防，CRS发生后通过中断贝林妥欧单抗治疗、根据分级评估给予糖皮质激素和IL-6受体阻滞剂治疗和（或）支持治疗，大多数患者在CRS消退后可继续贝林妥欧单抗的治疗[2],[14]。

低级别（1～2级）CRS无需暂停贝林妥欧单抗用药，可在密切监测相关指标及生命体征下给予对症治疗。对于严重（≥3级）CRS，3级需暂停给药，4级永久停药，要严密监测生命体征并迅速给予积极的治疗，根据临床指征给予糖皮质激素（首选地塞米松）和IL-6受体阻滞剂[42]。

（2）神经系统毒性：神经系统毒性的主要表现为颅神经受累的表现，如头痛、抽搐、言语障碍、意识障碍、意识模糊、定向障碍、协调障碍和平衡障碍等。神经系统毒性发生的确切机制目前尚不清楚，通过中断免疫治疗、给予糖皮质激素和（或）支持治疗，大多数神经系统毒性是可逆的[42]。在TOWER研究中，神经系统毒性发生率为45％，≥3级神经系统毒性发生率为9％，因神经系统毒性导致贝林妥欧单抗治疗中断率为6％，导致治疗终止率为4％。在中国的Ⅲ期单臂研究（316研究）中，≥3级神经系统毒性发生率为7％。神经系统毒性发生中位时间为输注开始第9天，大多数神经系统事件消退。神经系统毒性的最常见（≥10％）表现为头痛和震颤；神经系统毒性的特征因年龄组而异[2],[14]。

美国移植和细胞治疗协会（ASTCT）并未针对神经系统毒性给出处理方法和建议。在贝林妥欧单抗用于成人R/R ALL的临床试验中，所有≥3级的神经系统毒性均立即暂停给药并进行体格检查、生命体征监测及安全性相关实验室检查，根据患者情况给予地塞米松治疗，每日剂量至少24 mg/d，地塞米松减量时应于4 d内完成减量直至停药。支持治疗如癫痫发作的抗癫痫药物应由专科医师判断酌情使用[2],[42]。

七、结语

BiTE技术是相对成熟的双特异性抗体技术平台，目前首个获得批准上市用于临床治疗的BiTE分子为贝林妥欧单抗，为BCP-ALL患者的治疗带来了新选择。其以B淋巴系肿瘤高表达的CD19为靶点，利用自身T细胞对肿瘤的免疫作用，有效杀伤肿瘤细胞并清除MRD。在成人及儿童R/R B-ALL及MRD清除的治疗中均已显示出良好的疗效，安全性优于传统化疗。贝林妥欧单抗与减剂量化疗方案、新型TKI以及其他免疫治疗联合可能具有协同作用，大量联合方案的探索研究也在进行中。随着临床研究的深入和规范化应用的开展，贝林妥欧单抗必将更好地用于BCP-ALL的治疗，使更多患者获益。

**参与指导原则制定和讨论的专家：**

主审专家：马军、吴德沛、王建祥、沈志祥、胡豫、黄晓军

专家组成员（按姓氏拼音的先后顺序排名）：蔡真（浙江大学医学院附属第一医院）、陈苏宁（苏州大学附属第一医院）、杜欣（广东省人民医院）、方美云（大连大学附属中山医院）、高素君（吉林大学第一医院）、贡铁军（哈尔滨血液病肿瘤研究所）、韩悦（苏州大学附属第一医院）、何爱丽（西安交通大学第二附属医院）、侯健（上海交通大学医学院附属仁济医院）、胡豫（华中科技大学同济医学院附属协和医院）、黄晓军（北京大学人民医院）、纪春岩（山东大学齐鲁医院）、江明（新疆医科大学第一附属医院）、江倩（北京大学人民医院）、金洁（浙江大学医学院附属第一医院）、赖永榕（广西医科大附属第一医院）、李艳（中国医科大学附属第一医院）、李建勇（江苏省人民医院）、李娟（中山大学附属第一医院）、李军民（上海交通大学医学院附属瑞金医院）、梁爱斌（同济大学附属同济医院）、刘代红（解放军总医院）、刘启发（南方医科大学南方医院）、刘卓刚（中国医科大学附属盛京医院）、罗建民（河北医科大学第二医院）、马军（哈尔滨血液病肿瘤研究所）、马丽萍（中山大学孙逸仙纪念医院）、牛挺（四川大学华西医院）、宋永平（河南省肿瘤医院）、沈志祥（上海交通大学医学院附属瑞金医院）、孙自敏（中国科学技术大学附属第一医院、安徽省立医院）、王建祥（中国医学科学院血液病医院）、王季石（贵州医科大学附属医院）、王迎（中国医学科学院血液病医院）、魏辉（中国医学科学院血液病医院）、吴德沛（苏州大学附属第一医院）、肖志坚（中国医学科学院血液病医院）、徐雅婧（中南大学湘雅医院）、杨林花（山西医科大学第二医院）、张连生（兰州大学第二医院）、张苏江（上海交通大学医学院附属瑞金医院）、张曦（陆军军医大学第二附属医院）、张晓辉（北京大学人民医院）、赵东陆（哈尔滨血液病肿瘤研究所）、赵维莅（上海交通大学医学院附属瑞金医院）、周道斌（北京协和医院）、*周剑峰*（华中科技大学同济医学院附属同济医院）
